# Exploring maternal-fetal interface with in vitro placental and trophoblastic models

**DOI:** 10.3389/fcell.2023.1279227

**Published:** 2023-11-14

**Authors:** Xinlu Liu, Gang Wang, Haiqin Huang, Xin Lv, Yanru Si, Lixia Bai, Guohui Wang, Qinghua Li, Weiwei Yang

**Affiliations:** ^1^ School of Biosciences and Biotechnology, Weifang Medical University, Weifang, Shandong, China; ^2^ Department of Emergency, Affiliated Hospital of Weifang Medical University, Weifang, Shandong, China; ^3^ School of Public Health, Weifang Medical University, Weifang, Shandong, China

**Keywords:** placenta, trophoblast cells, trophoblast stem cells, organoids, *in vitro* models

## Abstract

The placenta, being a temporary organ, plays a crucial role in facilitating the exchange of nutrients and gases between the mother and the fetus during pregnancy. Any abnormalities in the development of this vital organ not only lead to various pregnancy-related disorders that can result in fetal injury or death, but also have long-term effects on maternal health. *In vitro* models have been employed to study the physiological features and molecular regulatory mechanisms of placental development, aiming to gain a detailed understanding of the pathogenesis of pregnancy-related diseases. Among these models, trophoblast stem cell culture and organoids show great promise. In this review, we provide a comprehensive overview of the current mature trophoblast stem cell models and emerging organoid models, while also discussing other models in a systematic manner. We believe that this knowledge will be valuable in guiding further exploration of the complex maternal-fetal interface.

## 1 Introduction

The placenta is an extra-embryonic organ that plays a crucial role in supporting and facilitating fetal development within the uterus. It serves as a vital conduit for oxygen, nutrients, and waste removal (Maltepe and Fisher, 2015). Impaired placental function can have profound implications on both maternal and fetal health, potentially leading to serious pregnancy complications (Silasi et al., 2010; Zhou et al., 2013; Mele et al., 2014; Liu et al., 2022). However, our current understanding of the human placenta remains limited due to the absence of functional experimental models. This constraint has impeded research efforts aimed at elucidating the etiology of placental disorders and developing effective therapeutic interventions. Therefore, establishing *in vitro* models can enhance our comprehension of intricate placental diseases while also enabling novel therapeutic strategies.

## 2 Human placenta

### 2.1 Macroscopic structure of the placenta

**FIGURE 1:**
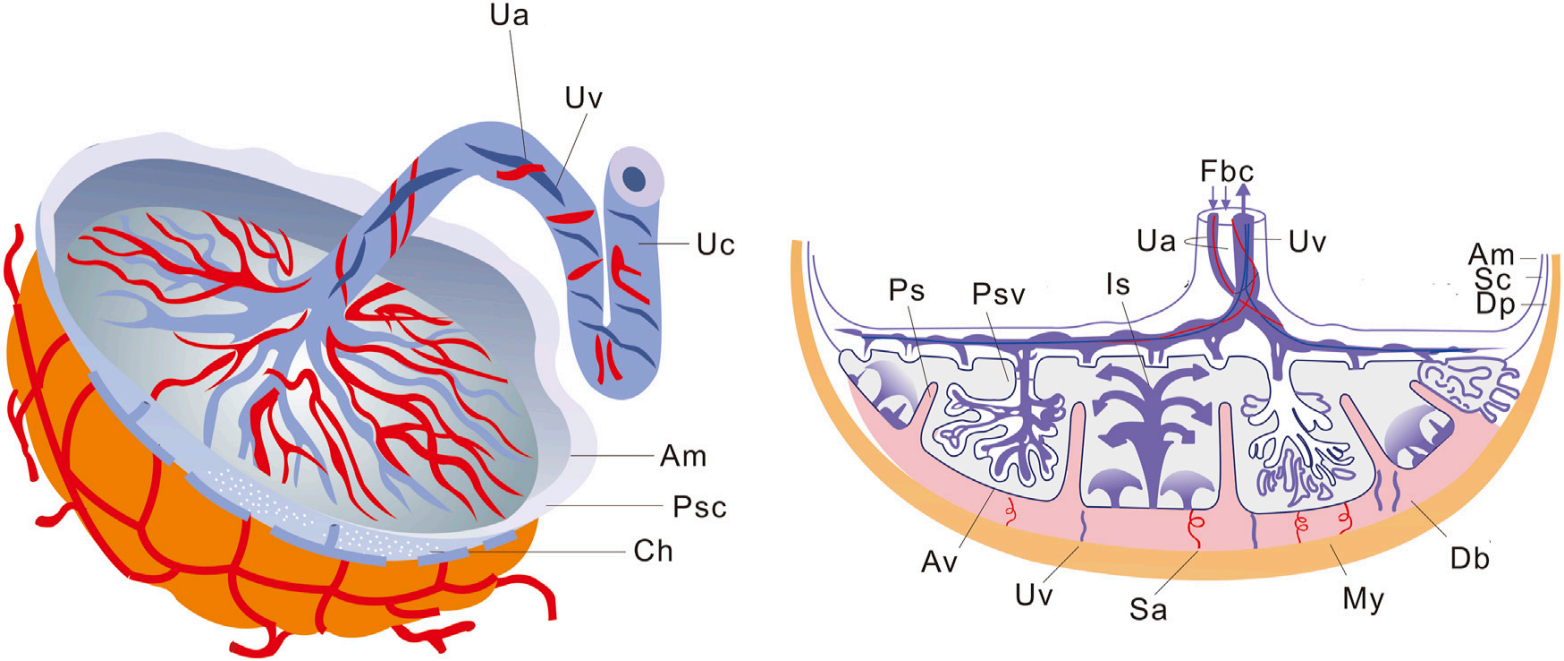
Schematic representation of placental structure. The macroscopic structure of the placenta is shown on the left, and the sagittal section of the placenta is shown on the right. *Ua*, Umbilical artery; *Uv*, Umbilical vein; *Uc*, Umbilical cord; *Am*, Amnion; *Psc*, Placental stem cells; *Ch*, Chorion; *Fbc*, Fetal blood circulation; *Is*, Intervillous space; *Ps*, Placental septum; *Psv*, Primary stem villus; *Sc*, Smooth chorion; *Dp*, Decidua parietalis; *Db*, Decidua basalis; *My*, Myometrium; *Sa*, Spiral arteries; *Av*, Anchoring villus.

The placenta, which originates from both the embryonic and maternal endometrium, plays a crucial role in facilitating material exchange between the fetus and the mother (Covarrubias et al., 2023). It is disc-shaped and remains embedded in the inner wall of the uterus until delivery, when it is expelled due to myometrial contractions (Leiser and Kaufmann, 1994; Wheeler and Oyen, 2021). Figure 1 illustrates that the placental thickness is greater at its center and thinner at its margin. The smooth side facing inward is called the chorionic plate, to which the umbilical cord attaches, connecting each villus. On the rough basal plate, there are dendritic villus, which constitute a major component of the placenta. These finger-like blood vessels allow for maternal blood flow through the intervillous space, enabling material exchange across their intricate network, from mother to fetus, while waste products are excreted.

### 2.2 Major components of the placenta

The placenta is a complex organ consisting of three main components: the amnion, chorion, and decidua basalis (Huppertz, 2008). The amniotic membrane serves as both the innermost layer of the placenta and umbilical cord, as well as the fetal part of the placenta. It has a smooth texture without any vascular or lymphatic structures and exhibits a certain level of elasticity (Chuva de Sousa Lopes et al., 2022). Initially, the amniotic membrane was attached to the edge of the embryonic disc. As it expanded within the amniotic cavity, it surrounded the ventral surface of the embryo’s body stalk, resulting in the formation of the primitive umbilical cord (Huppertz, 2008).

The decidua basalis, although a small portion of the term placenta, plays a crucial role in immune regulation as it is an integral component of maternal tissue (Zhang and Wei, 2021; Jin et al., 2023). The decidua basalis surface is coated with a layer of trophoblast cells originating from the fixed chorionic villus. This bottom metaphase, along with the trophoblast cells, constitutes the base of the chorionic interstices, known as the decidua plate. From this plate, several decidua intervals extend towards the chorionic membrane, dividing the maternal side of the placenta into approximately 20 visible maternal lobes.

The chorion, which is the main component of the fetus, is covered by the chorionic membrane. This membrane directly contacts the endometrium and other accessory structures. Within the intervillous space, there are villus filled with maternal blood and basal decidua-embedded villus (Huppertz, 2008). The development of these villus enhances the contact between the chorion and uterine decidua, allowing for material exchange between the embryo and mother (O'Brien and Wang, 2023).

The villus in contact with the decidua basalis are well developed due to the abundance of nutrients and are referred to as phyllodes chorion. The ends of the villus that are suspended in the intervillous space, filled with maternal blood, are known as free villus, while those growing into the decidua basalis are called fixed villus. The fetal lobe is partially separated into maternal lobes, with each maternal lobe containing several fetal lobes. Each fetal lobe has its own spiral artery for blood supply (Gruenwald, 1977).

The uterine spiral artery passes through the decidual plate and enters the maternal lobe. Material exchange between the mother and the fetus occurs at the villus of the fetal lobule (Pijnenborg et al., 2006). This indicates that fetal blood exchanges with maternal blood in the intervillous space through the umbilical artery until it reaches the capillary network in the villus. It is important to note that fetal blood and maternal blood do not communicate directly (Cindrova-Davies and Sferruzzi-Perri, 2022). In a well-structured placenta, maternal nutrients enter the fetal blood through six layers of tissue. These layers include the endothelial cell layer of the maternal microvascular wall, the connective tissue layer, the epithelial layer of the endometrium, the germinal layer of the fetal chorionic membrane, the connective tissue layer of the fetal villus core, and the endothelial cell layer of the fetal microvascular wall.

### 2.3 Development of the human placenta

**FIGURE 2:**
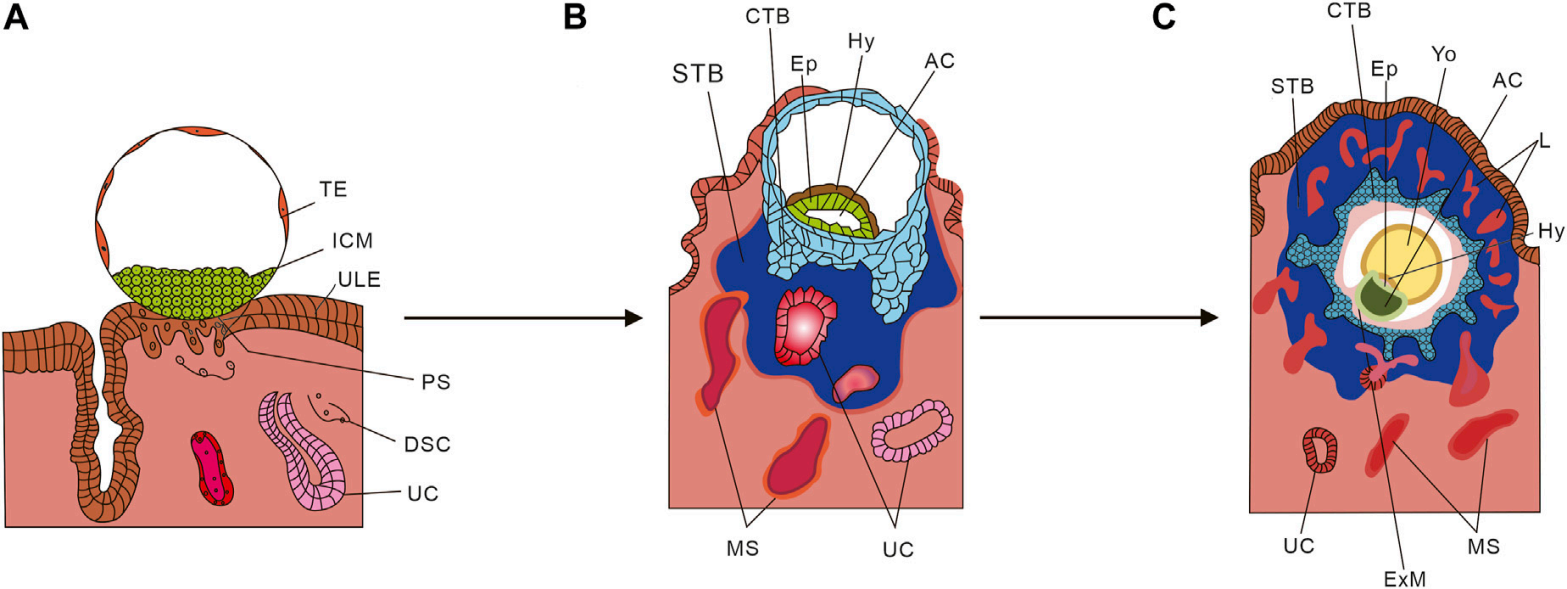
Schematic diagram of placental development. **(A)** Human blastocyst implanting into the pregnant uterus. **(B)** Development of the first placental structures and the embryonic disc. **(C)** Formation of primary villus and yolk sac. *TE*, Trophectoderm; *ICM*, Inner cell mass; *ULE*, Uterine luminal epithelium; *PS*, Primitive syncytium; *DSC*, Decidual stromal cell; *UC*, Uterine capillary; *Hy*, Hypoblast Ep; *STB*, Syncytiotrophoblasts; *CTB*, Cytotrophoblast; *MS*, Maternal blood sinusoid; *Yo*, Yolk sac; *L*, Lacunae system; *AC*, Amniotic cavity; *ExM*, Extraembryonic mesoderm. (From Cindrova-Davies T, Sferruzzi-Perri AN. Human placental development and function. Semin Cell Dev Biol. 2022 Nov; 131:66-77. doi: 10.1016/j.semcdb.2022.03.039. Epub 2022 Apr 4. And make some changes).

The development of the human placenta involves a collaborative interaction between the trophoblast layer of the placenta and the maternal endometrium. Trophectoderm (TE) is the initial cell type that gives rise to all trophoblast cells and it starts developing 4–5 days after fertilization. Once the TE separates from the inner cell mass (ICM), the ICM proceeds to form an embryo. As depicted in Figure 2A, the first stage of placental development involves the implantation of a human blastocyst into the uterus of the pregnant individual. This process typically begins around 6–7 days after fertilization, during which the wall of the blastocyst differentiates into the placenta upon implantation. Figure 2B depicts the initial development of the primary structures of the placenta. Once the embryo attaches to the uterus, the trophoblast cells infiltrate the endometrium (Bentin-Ley et al., 2000; Illsley et al., 2020) and undergo primary syncytiotrophoblast (STB) formation (Shao et al., 2021). During this stage, the inner cell mass (ICM) transforms into a bilaminar epithelial structure comprising of the epiblast (Ep) and hypoblast (Hy; also known as primitive endoderm). Subsequently, a significant number of STB infiltrate between the endometrial cells, leading to the transformation of the endometrium into a specialized tissue known as decidua (Ruiz-Magaña et al., 2022). Trophoblast cells that do not come into contact with the intima do not undergo the primary integration process and become cytotrophoblast (CTB) (Shao et al., 2022). The period between 7 and 8 days after fertilization is referred to as the prelacunar period of placental development. On the 8th day after fertilization, vacuole-like structures start to appear in the hypertrophic STB at the implantation site. These vacuoles gradually enlarge and merge with each other, forming a complete lacunar space, indicating the transition of placental development into the lacunar period (Enders, 1995).

From day 8 to day 13 after fertilization, the cytotrophoblast (CTB) in the blastocyst undergoes proliferation and gradually merges with the syncytiotrophoblast (STB), resulting in its thickening and eventual envelopment of the entire blastocyst with lacunae. During this process, the CTB proliferates in layers to form trophoblast columns, while the STB penetrates the maternal uterine capillary (UC), giving rise to discontinuous maternal blood sinus (MS). As depicted in Figure 2C, around day 14 after fertilization, the CTB completely infiltrates the STB and forms the cytotrophoblast sheath, which signifies the onset of the villous stage of placental development. In this period, lamellar or columnar STB extend branches composed of CTB, known as primary villous stems, which branch out to form a dendritic complete villous structure. At this point, the aforementioned cavity structure is officially referred to as the intervillous space (Cindrova-Davies and Sferruzzi-Perri, 2022).

The cytotrophoblast sheath initiates a process of deep invasion and migration when it comes into contact with the maternal decidua (Gude et al., 2004). At this stage, the CTB separates from the villous structure and transforms into extravillous trophoblast (EVT). The EVT that penetrate the deep layer of endometrium are referred to as interstitial EVT (iEVT), while the remaining portion of EVT cells is known as endovascular EVT (enEVT) (Brkić et al., 2018). During early placental development, enEVT infiltrates into the spiral arteries of the endometrium and gradually remodels by interacting with endothelial cells (Sato, 2020). The invasion of placental trophoblast cells into the endometrial matrix and subsequent reconstruction of uterine spiral arteries are crucial physiological processes for maintaining a successful pregnancy. They also play a pivotal role in establishing utero open blood circulation (Albrecht and Pepe, 2020).

During pregnancy, the CTB undergoes continuous development, leading to the formation of a fully developed STB. The STB envelops the surface of placental formation and establishes a selectively permeable barrier between the maternal and fetal compartments. Additionally, it serves as a crucial structural link connecting these compartments.

The placenta has two main functions in establishing and maintaining an optimal growth environment for the embryo. Firstly, it facilitates the circulation and exchange of materials between the mother’s uterus and the fetus. This involves the transfer of substances between the fetal blood vessels and the maternal blood through the semi-permeable barrier created by the villus. Secondly, trophoblast cells invade and remodel the uterine blood vessels, further enhancing this complex process (Staff et al., 2022). The placenta also plays a role in maintaining the growth environment by ensuring the fetal growth microenvironment and promoting maternal-fetal immune tolerance. This is achieved through the maintenance of decidualization of the endometrium and the control of uterine contractile response by progesterone (Amini et al., 2019). The placental chorion has an immunomodulatory effect, which includes inhibiting excessive inflammation after blastocyst implantation, regulating uterine natural killer cells (Xie et al., 2022), and promoting the proliferation of regulatory T cells (Paolino et al., 2021).

The placenta plays a crucial role in pregnancy by synthesizing various hormones, enzymes, and cytokines. It produces hormones similar to those produced by the pituitary and ovary to support pregnancy. During early pregnancy, the placenta secretes human chorionic gonadotropin (hCG), which is similar to luteinizing hormone (LH) and helps maintain the development of the ovary’s corpus luteum. It also promotes the secretion of estrogen and progesterone by the corpus luteum. Additionally, the placenta secretes human placental prolactin (hPL), a hormone that stimulates the growth of both the placenta and fetus, as well as the development of the maternal mammary gland. The placenta, through the secretion of hCG, hPL, progesterone, and alpha-fetoprotein synthesized by the fetal liver, inhibits the maternal immune response, allowing normal fetal development in the uterus without triggering an immune reaction. Furthermore, the placenta possesses various properties such as anti-inflammatory, antibacterial, and anti-scar activities. These properties, combined with the common practice of discarding the placenta after childbirth, have facilitated its use in cell therapy research, regenerative medicine, and *in vitro* organ-tissue related studies. Overall, the placenta is a dynamic and multifaceted organ that supports the development and survival of the embryo and fetus.

The placenta is capable of synthesizing various hormones, enzymes, and cytokines. It secretes hormones similar to those produced by the pituitary and ovary to support pregnancy. Additionally, it produces short-range hormones similar to peptides from the hypothalamus. During early pregnancy, the placenta secretes hCG (Stenman et al., 2006; Barrera et al., 2008), which is similar to luteinizing hormone (LH) and helps maintain the development of the ovary’s corpus luteum. It also promotes the secretion of estrogen and progesterone by the corpus luteum. Another hormone secreted by the placenta is hPL, which promotes the growth of the placenta, fetus, and maternal mammary gland (Walker et al., 1991). The placenta also secretes progesterone (Kolatorova et al., 2022) and alpha-fetoprotein (Melamed et al., 2023), synthesized by the fetal liver, which inhibit the maternal immune response, allowing normal development of the fetus in the uterus without causing rejection. Furthermore, the placenta is a dynamic and multifaceted organ that supports the development and survival of the embryo and fetus (Tan et al., 2023). It possesses anti-inflammatory properties (Aggarwal et al., 2019), as well as antibacterial and anti-scar activity. These characteristics, combined with the common practice of discarding the placenta after childbirth, have facilitated its use in cell therapy research, regenerative medicine, and *in vitro* organ-tissue related studies.

## 3 Cell models

Trophoblast cells, which are epithelial cells in the placenta, play a crucial role in fetal growth and development throughout pregnancy. Abnormal trophoblast differentiation has been linked to complications such as miscarriage (Wang et al., 2021), preeclampsia (Huppertz, 2018), and fetal growth restriction. To understand placental disorders better, it is essential to have a comprehensive understanding of trophoblast structure and function during pregnancy. However, studying trophoblast cells has been challenging due to the lack of reproducible and widely used model systems. This has hindered significant advancements in this field. Fortunately, there has been recent progress in our ability to model this critical cell type at the maternal-fetal interface.

### 3.1 Placental derived TSCs models

Trophoblastic stem cells (TSCs) are derived from the trophectoderm, which corresponds to embryonic pluripotent stem cells (ESCs) derived from the inner cell mass of blastocysts. These TSCs have the ability to differentiate into various placental trophoblast cells during subsequent development, making them an invaluable *in vitro* model for studying the molecular mechanisms of placental development. Previous studies have successfully cultured mouse TSCs, providing a powerful *in vitro* platform for investigating the molecular mechanisms and functions of mouse embryonic development (Kidder, 2020; Ohinata et al., 2022; Seong et al., 2022). TSCs were first isolated and cultured from mouse extraembryonic ectoderm (ExE) (Tanaka et al., 1998). When mouse ExE cells were cultured in the presence of fibroblast growth factor 4 (FGF4) (Roberts and Fisher, 2011), highly proliferative epithelial colonies expressing Errb, Cdx2, Fgfr2, and Eomes were generated. The establishment of a mouse TSCs model has enabled researchers to comprehend the key gene network (Oct4, Ets2, Fgf4, Elf5) that maintains mouse TSCs in an undifferentiated state or identify key regulators involved in trophoblast cell differentiation (Tfap2c, Eomes, Cdx2, Gata3) (Kuijk et al., 2008; Rossant, 2008; Chen et al., 2021). It is crucial to establish a reliable model of human trophoblast cells in order to compare differences in early developmental embryos from human embryos using the same conditions.

In 2018, Okae et al. (2018) made a significant advancement in trophoblast cell research by successfully isolating TSCs from blastocysts and early pregnancy villus. These cells can be cultured for a long time for purification or cryopreserved for future use. The researchers achieved this by activating the Wnt signaling pathway (Sonderegger et al., 2010) and inhibiting Rho-associated protein (ROCK), histone deacetylase, and TGF-β. The TSCs also expressed trophoblastic markers such as TEAD4, GATA3, TP63 and ELF5 (Kuijk et al., 2008; Rossant, 2008; Chen et al., 2021). Additionally, they demonstrated the ability to differentiate into hCG^+^ STB (Horii et al., 2019) and HLA-G^+^ EVT (Xu et al., 2020).

The recent study by Vento-Tormo et al. (2018) utilized single-cell RNA sequencing to examine the cellular heterogeneity in early pregnancy and term placentas. Consequently, there are variations between TSCs models derived from early pregnancy and those derived from term placentas. Wang et al. (2022) established an effective method for obtaining TSCs from term placenta. Their research revealed that the induction efficiency of CTB is influenced by the functional antagonism between the placental transcription factor GCM1 and the stemness regulator ΔNp63α. This antagonism reduces the transcriptional activity of GCM1, while GCM1 inhibits ΔNp63α oligomerization and autoregulation. GCM1 serves as a major transcriptional regulator for trophoblast cell differentiation, promoting fusion, invasion, and hormone secretion of trophoblast cells. It is crucial for the differentiation of STB and EVT (Liang et al., 2010; Cheong et al., 2015; Jeyarajah et al., 2022). ΔNp63α, an isoform of the p63 transcription factor, exhibits high expression in CTBS (Lee et al., 2007; Li et al., 2013). By inducing ΔNp63α activity, inhibiting GCM1 expression through EGF/CASVY, and further disrupting GCM1 expression through hypoxia, the researchers successfully transformed CTB cells from term placenta into TSCs. This breakthrough provides a new avenue for investigating pregnancy diseases and trophoblast cell differentiation.

Significant progress has been made in TSCs research, and it is now possible to obtain equivalent cell lines from human blastocysts for studying the development and function of human trophoblast cells. However, there are still some challenges that need to be addressed. Firstly, ethical and legal issues, as well as potential diseases associated with the use of these cells, may limit their applicability, similar to primary cells obtained from these tissue sources. Secondly, although the characteristics of these cells, such as ITGA6 expression (Wu et al., 2022), suggest their origin from cytotrophoblast blasts, their exact location in the placenta remains unknown. Therefore, further studies are required to establish a comprehensive and systematic model.

### 3.2 ESCs and pluripotent stem cells (PSCs) derived TSCs models

ESCs are totipotent cells derived from early mammalian embryos that can proliferate indefinitely *in vitro* and remain undifferentiated (Evans and Kaufman, 1981). Extensive research has been conducted on ECSs derived from mice, which have significantly contributed to our understanding of various molecular mechanisms (Baribault and Kemler, 1989; Sato and Nakano, 2001; Lau et al., 2022). Additionally, non-human primate ES cell lines have been utilized as accurate *in vitro* models for studying the differentiation of human tissues (Lester et al., 2004; Kishimoto et al., 2021). However, due to ethical and practical reasons, human relevant models have not been extensively explored. In 1998, Thomson et al. (1998) reported the development of pluripotent cell lines derived from human blastocysts. These cell lines exhibited normal karyotypes and expressed high levels of telomerase activity, as well as cell surface markers characteristic of primate ESCs. Although the characterization of other early cell lineages was not performed, these cells retained their developmental potential even after 4–5 months of *in vitro* proliferation. They were capable of forming trophoblasts and derivatives of all three embryonic germ layers, including the intestinal epithelium (endoderm), cartilage, bone, smooth muscle, and striated muscle (mesoderm), as well as neuroepithelium, embryonic ganglia, and stratified squamous epithelium (ectoderm). These cell lines hold immense promise in the fields of human developmental biology, drug discovery, and transplantation medicine (Gearhart, 1998). However, they also give rise to scientific hopes and pose legal issues (Marshall, 1998).

Then Ezashi et al. (2012) utilized human embryonic stem cells (hESC) as a model to investigate trophoblast differentiation. They exposed both hESCs and iPSCs to the growth factor BMP4 (Xu et al., 2002; Koel et al., 2017) in order to generate trophoblast lineage cells. This model system allowed them to explore the initial events that determine the specification of trophoblasts and their subsequent transformation into more specialized lineages, as well as the role of oxygen in this differentiation process. Additionally, they extensively described the significant role of BMP4 in differentiating ESCs and PSC into trophoblast lineages (Ezashi et al., 2005; Das et al., 2007; Karvas et al., 2018). The original hESC model, which employed only BMP4 without FGF2, has been widely used to study trophoblast development. However, there have been some concerns suggesting that this differentiation primarily leads to mesoderm rather than trophoblast. In 2013, R. Michael Roberts and his team demonstrated that hesCs grown in mouse embryonic fibroblast medium containing BMP4 but no FGF2 rapidly transformed into epithelial cells expressing more trophoblastic markers and fewer mesodermal markers. In response to doubts regarding whether the BMP4/hESC *in vitro* model primarily differentiates into mesoderm rather than trophoblast (Bernardo et al., 2011), they indicated that it can indeed be utilized to study the emergence and differentiation of trophoblast cells. This study revealed that optimal trophoblast differentiation can be achieved by maximizing BMP4 signaling while minimizing MEK/ERK signaling (Amita et al., 2013). Subsequently, in 2015, they reported a new study on BMP4, which demonstrated that transient exposure to BMP4 for 24–36 h increased the potency of PSCs. When combined with ACTIVIN signaling inhibitors A83-01 and FGF2, this led to the acquisition of unique stem cell phenotypes from hESCs and iPSCs (Yang et al., 2015; Roberts et al., 2018). It has been demonstrated that BMP4 can induce human hPSCs to a self-renewing alternative state that allows trophoblast development, with implications for the regulation of lineage determination in early embryos.

In 2016, Horii et al. (2016) proposed a reproducible two-step protocol to differentiate into hPSCs dual-energy TSCs, followed by redifferentiation into fully functional trophoblast cells. The protocol involved inducing hPSCs to differentiate into CDX2+/p63+CTB stem cells using a specific medium containing BMP4. These CTB stem cells demonstrated self-renewal capacity and the potential to differentiate into STB and EVT cells, as confirmed by marker expression, hormone secretion, and invasive capacity. To evaluate the applicability of hPSCs-derived CTBs, the researchers successfully replicated the delayed CTB maturation and impaired STB differentiation observed in trisomy 21 syndrome (T21) (Gerbaud et al., 2019) hPSCs. Overall, their study emphasizes that hPSCs provide a reliable model for studying human trophoblast development and accurately replicating trophoblast cell differentiation defects. In 2022, the researchers used a new protocol to generate functional TSCs from primed hPSCs. They followed the previously established two-step protocol to differentiate induced hPSCs into functional trophoblasts and then switched to the newly developed TSCs medium to generate authentic TSC (Soncin et al., 2022). Through sequencing and transcriptome analysis, they found that these cells resembled placenta and naive hPSC-derived TSCs, and exhibited similar differentiation potential both *in vitro* and *in vivo*. The new scheme offers a simpler approach that can be applied to a wide range of existing hPSCs, including induced PSCs derived from patients with known birth outcomes.

Mammalian embryonic development initiates with the formation of the inner cell mass and trophectoderm, although the exact mechanism remains unclear. In a study conducted by Kobayashi et al. (2022) in 2022, it was discovered that naive human embryonic stem cells (hESCs) have the ability to transdifferentiate into TSCs, whereas priming hESCs cannot. This discrepancy was attributed to the highly active primate-specific miRNA cluster (C19MC) located on chromosome 19 of naive hESCs, which triggers post-HES silencing. Further investigation revealed that C19MC is crucial for maintaining hTSC cells, and the activation of hESCs cells can generate hTSCs cells. These findings demonstrate that the activation of C19MC equips hESCs cells with the potential to differentiate into trophoblast cell lineages, thereby providing a significant molecular mechanism for the development of TSCs models.

Table 1 presents trophoblast cell models developed in recent years, summarizing their sources and direction of differentiation, along with the main methods used for their construction. Simulating real-time pathophysiological changes *in vivo* remains a challenge for cell models, despite the attention garnered by TSCs models obtained from different sources and using different methods (Li et al., 2019; Castel et al., 2020; Cinkornpumin et al., 2020; Dong et al., 2020; Mischler et al., 2021; Wei et al., 2021; Castel and David, 2022; Dong and Theunissen, 2022; Tan et al., 2022). While traditional 2D cell culture has been convenient and widely adopted for many years, it lacks the ability to closely mimic *in vivo* conditions. In contrast, 3D tissue culture offers a physiologically relevant environment that more closely resembles *in vivo* conditions. As a result, researchers have started exploring 3D environments to observe physiological and pathological changes in cells and organs.

**TABLE 1:** Representive Trophoblastic Models in recent years.

The author	Date	Source	Differentiation direction	Main methods, etc.
Gaël Castel et al.	2020	Somatic cell and pluripotent stem cell	Blastocysts and first trimester CTB	Reprogramming or cell fate conversion
Jessica K Cinkornpumin et al.	2020	Naive hESCs	HTSCs derived from human placenta or blastocys	Transdifferentiation
Chen Dong et al.	2020	Naïve hPSCs	Extravillous and STB	Differentiation
Yanxing Wei et al.	2021	Primed PSCs	TSCs	Chromatin accessibility dynamics and histone modifications
Zhuosi Li et al.	2019	hTSCs	STB and EVT	Micromesh culture technique
Adam Mischler et al.	2021	hPSCs	Two distinct stem cell types of the trophectoderm	Derivative of differentiation
Gaël Castel et al.	2022	hPSCs	CTB, EVT	Reprogram somatic cells
Jia Ping Tan	2022	Fibroblasts	hTSCs	Reprogramming
Dong C et al.	2022	Human Naïve PSCs	hTSCs	Derivation of cells

### 3.3 Primary trophoblasts

Primary cells are obtained from living tissues through isolation methods and are not immortalized like cell lines. They retain their original genetic characteristics and closely resemble the growth status of cells *in vivo*. This makes them ideal for experimental research, including drug testing, cell differentiation, and transformation (Anvarian et al., 2019).

In recent decades, studies on primary trophoblast cells have been conducted using placental tissue from various stages of pregnancy. The most easily accessible samples are obtained after normal full-term pregnancies and late-gestation deliveries. However, it is difficult to determine whether placental pathology is a cause or a consequence of EVT function, which plays a crucial role in the development of pregnancy disorders such as preeclampsia during early placental formation. The invasiveness and motility of EVT are significantly reduced at term. Quenby et al. (2004) investigated the impact of heparin and aspirin on EVT; however, their study did not establish any correlation between this effect and markers such as PP13 (Piskun et al., 2022), PIGF (Author Anyonoums, 2019; Wang et al., 2020), PAPP-A (Conover and Oxvig, 2023), or any other known predictive markers for preeclampsia (Townsend et al., 2019). Therefore, researchers are still searching for new molecules that can serve as markers for EVT. Consequently, the *in vitro* formation of STB during different developmental processes of trophoblast cells can effectively be studied using human full-term placenta.

The isolation of chorionic trophoblast cells has been conducted in multiple laboratories, followed by freezing and storage for various programs (Sagrillo-Fagundes et al., 2016). In the 1980s, a traditional tryptic digestive procedure was established and has been utilized since then. Kliman et al. (1986) later introduced the Percoll gradient procedure, which significantly enhanced the purity of chorionic trophoblast cells. Additionally, the use of magnetic beads allowed for further purification of these cells (Douglas and King, 1989). It was not until 2002 that Guilbert et al. (2002) (Huppertz et al., 1999) and Tannetta et al. developed methods to isolate CTB from mononuclear ensemble fragments.

However, Tannetta et al. (2008) demonstrated that isolated primary trophoblast cells no longer proliferate in culture. They tested the effects of antioxidants vitamin C and vitamin E on cultured primary trophoblast cells and found that after 96 h of culture, the cells underwent apoptosis, as evidenced by the loss of pase9 and Caspase3. Additionally, there was an increased shedding of CTB cell debris accompanied by apoptosis. Vitamins C and E were able to prevent apoptosis and shedding of CTB fragments, but they also resulted in decreased fusion of isolated trophoblast cells. Therefore, further studies on effective *in vitro* model systems are still pending.

### 3.4 Human placental passage cell lines

Human trophoblast cell lines BeWo, JEG-3, and JAR have been extensively utilized in studying the functions of trophoblast cells when human samples are not available. BeWo is an endocrine cell line derived from malignant gestational choriocarcinoma tissue (Pattillo and Gey, 1968). JEG-3 is one of the six different cell lines derived from choriocarcinoma clones, established serially (Kohler and Bridson, 1971). JAR, on the other hand, was established from placental trophoblast cell tumors. These cell lines exhibit similar foreign body metabolism enzymes to human placenta, such as CYP1A1 and CYP19 (Wójtowicz et al., 2011), making them suitable for investigating placental hormone secretion, uptake, efflux, and metabolism of exogenous foreign substances by placental cells.

JEG-3 and BeWo cell lines are commonly used as trophoblast cell models in early pregnancy. Champoothiri et al. (Nampoothiri et al., 2007) studied the protein profile of the BeWo cell line induced by forskolin. Additionally, the JEG-3 cytotrophoblast cell line can serve as a model for studying the function of isoforms of ADAMTS (a disintegrin and metalloproteinase with thrombospondin repeats) in epithelial cell proliferation (Beristain et al., 2011). The BeWo b30 cell line is commonly used to investigate placental barrier function at the cellular level. It forms tightly polarized monolayer cells on Transwell culture plates, although it has been associated with P-gp (Mark and Waddell, 2006). Furthermore, JEG-3 and BeWo b24 cell lines have the potential to develop monolayer polarized tight junctions, making them promising models for *in vitro* studies on placental drug transport after optimizing the appropriate conditions (Ikeda et al., 2011; Crowe and Keelan, 2012).

The BeWo cell line is widely used as a model for choriotrophoblast cells. It shares many characteristics with choriotrophoblast cells, such as the ability to fuse and integrate. However, due to the complex origin of BeWo cells, it is unclear which stage of pregnancy they represent. Additionally, BeWo cells can cause different changes in cell lines and varying fusion rates. Therefore, it is important to compare BeWo cells with primary trophoblast cells at different stages of pregnancy. Comparisons between BeWo and JEG-3 cells showed that they had the same DNA profile and similar secretory activity. However, BeWo cells were found to be highly sensitive to forskolin-induced syncytial formation (Vargas et al., 2009; Schneider et al., 2011), while JAR cells retained many of the morphological and endocrine features observed in human trophoblasts. Furthermore, the expression of certain proteins in the immortalized cell lines did not align with that of primary cells. For example, the expression of P-gp in BeWo, JAR, and JEG-3 cell lines was lower compared to primary trophoblasts isolated from term placentas (Evseenko et al., 2006). The expression of BCRP in BeWo and JAR cells was similar to that in human placenta, except for JEG-3. BeWo and JAR cells exhibited higher levels of ABCC2 and lower levels of ABCC1 compared to primary trophoblasts (Wolfe, 2006).

Although the cell line has the advantage of being repetitive and stable, the method of changing telomerase and viral transformation also leads to alterations in gene expression (Shiverick et al., 2001), thereby necessitating further improvement in the cell line model. However, these cell models still have certain limitations, such as their inability to simulate real-time pathophysiological changes *in vivo*. While traditional 2D cell culture has been widely adopted for many years due to its convenience, 3D tissue culture offers a more physiologically relevant environment that closely mimics *in vivo* conditions. As a result, researchers have started exploring 3D models to observe physiological and pathological changes in cells and organs.

## 4 Trophoblast organoid models

### 4.1 Introduction to organoids

In recent years, organoids have become indispensable tools for studying disease mechanisms (Corrò et al., 2020). They utilize various types of stem cells, including ESCs, induced pluripotent stem cells (iPSCs), and adult stem cells (ASCs), to self-organize in a 3D culture environment through cell sorting and space restriction (Hagen and Hinds, 2019). Organoids are small, simulated organs that closely resemble real organs in structure and function, although they do not possess full functionality. They exhibit remarkable self-renewal and self-organization capabilities and can replicate the organ-specific functions of the tissues they originate from (Hagen and Hinds, 2019).

The concept of 3D organoid culture dates back to the early 20th century, with Wilson et al. first demonstrating the self-organization and regeneration ability of isolated sponge cells to become complete organisms in 1907 (Wilson, 1907). However, in 2009, Hans Clevers and his team pioneered the intestinal organoid culture system, marking the beginning of a “new era” of organoid technology (Barker et al., 2009). Since then, organoids have revolutionized various fields, including basic research, medicine, and regenerative medicine. Their ability to faithfully reproduce complex 3D structures, different cell types, and specific organ functions makes them valuable models for studying organ development and disease. As a result, they hold great potential for a wide range of biological and medical applications (Rossi et al., 2018; Hofbauer et al., 2021).

### 4.2 Trophoblastic organoids (CTB-ORGs) models

In 2018, the groundbreaking research conducted by Turco et al. (2018) led to the establishment of CTB-ORGs. This model provides a powerful tool for studying the complex interactions between maternal and fetal cells during human placenta formation. The objective of this study is to effectively replicate the dual differentiation pathways of early placental trophoblast cells, specifically migration and infiltration. By doing so, it establishes a platform for observing and understanding the interaction between early placental development and trophoblast cells.

First, the researchers studied the Wnt and MAPK signaling pathways during the 6–8 weeks of gestation. They identified the basal medium components, including epidermal growth factor (EGF) (Cheng et al., 2022) and fibroblast growth factor 2 (FGF-2) (Martinez-Fierro et al., 2022), and enzymatically digested the placenta during early pregnancy. EpCAM-labeled trophoblast cells, which are a specific subtype of trophoblast cells, were obtained. These labeled trophoblast cells were then seeded in Matrigel drops rich in medium to support the stable growth of trophoblast cells (Zhao et al., 2022). In this study, proliferating EpCAM^+^ CTB were successfully isolated from first-trimester villus. Subsequently, human first-trimester trophoblast organoids were generated by activating the Wnt signaling pathway and inhibiting the TGF-β signaling pathway (Ye et al., 2022). Transcriptome microarray analysis revealed that the organoids exhibited similar transcriptome and methylation profiles to primiparous placentas. Mass spectrometry showed that the hormones and proteins secreted by the villus in the organoids were similar to those secreted by the placenta. The origin of the cells was confirmed by HLA typing, while their identity was verified according to specific criteria for four trophoblast cell types (Lee et al., 2016).

Turco et al. (2018) successfully generated human trophoblast organoids using villous trophoblast cells (VCT) and syncytial trophoblast cells (SCT). These organoids closely resemble the *in vivo* villous placenta in terms of anatomy and function, presenting complex 3D structures. Additionally, HLA-G positive EVT, which exhibited strong invasion ability in a 3D environment, was also identified during the experiment. The *in vitro* organoid model offers a significant advantage as it can replicate the complex placental structure and produce both STB and EVT. This model facilitates the study of mother-to-child transmission of xenobiotics, drugs, pathogens, proteins, and hormones from STB. Consequently, these trophoblastic organoids hold great promise in understanding maternal-fetal interactions post-implantation and exploring maternal physiological changes in metabolism and hormonal regulation during pregnancy.

However, the study of placental development has been stagnant due to the lack of models that can autonomously renew and differentiate, such as STB and EVT. One of the challenges is accurately reproducing the human trophoblast model of early placental development (Ma et al., 2023). In 2018, Haider et al. (2016) developed an organoid model with autonomous differentiation and renewal ability using purified CTB cells from early pregnancy. This model provides a valuable foundation for understanding the initial development mechanism of trophoblast cells, as well as cell proliferation and differentiation. Haider et al. (2016) cultured purified VCT extracted from early primiparous pregnancy placental tissue in Matrigel, supplemented with various growth factors and signal transduction inhibitors like A83-01 and GSK-3α/β. Within a few days of culture with these factors, small cell clusters rapidly formed and developed into 16 different organoid cultures with 100% derivation efficiency in 2–3 weeks. These organoids could be stably passaged for 5 months. CTB-ORGs, which are the organoids, express markers of trophoblast stemness and proliferation like ELF5 (Lee and Ormandy, 2012), CDX2 (Nishioka et al., 2009), TEAD4 (Saha et al., 2020; Stamatiadis et al., 2022), GATA3 (Chiu and Chen, 2016), and AP-2a (Orso et al., 2007). They have been identified and gene sequenced, confirming their long-term expansion ability and similarity to primary CTBs in terms of gene expression.

The presence of EGF (Cheng et al., 2022) and A83-01 was found to be essential for the long-term expansion of CTB-ORGs. On the other hand, the absence of Wnt inducers (R-pondin and CHIR99021) promoted the growth and differentiation of trophoblast cells. This suggests that WNT signaling plays a crucial role in organoid formation by controlling self-renewal, lineage delineation, and differentiation. Exogenous stimulation of Wnt signaling can maintain the growth of CTB-ORGs. Additionally, it was observed that the withdrawal of R-spondin and CHIR99021 led to a transient increase in cyclin A expression, promoting the growth of trophoblast cells and the development of proliferative NOTCH1^+^ progenitor cells. These findings provide a valuable molecular mechanism for future research.

**FIGURE 3:**
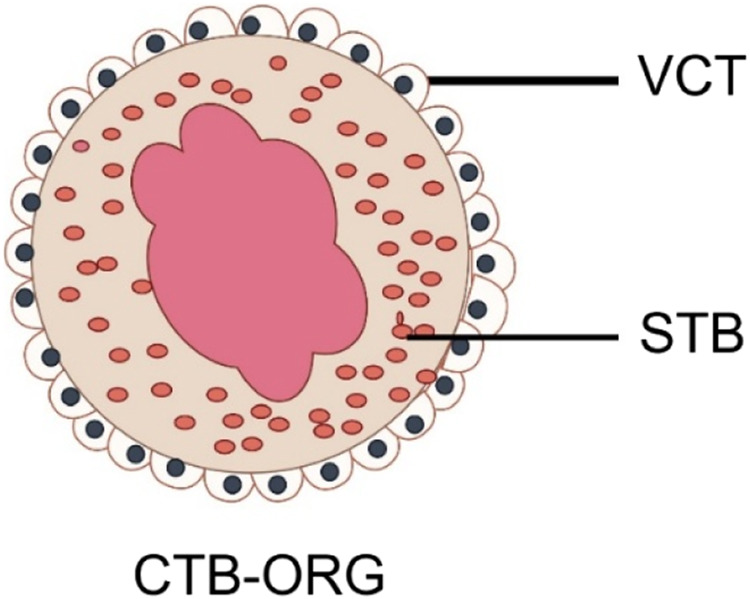
Schematic diagram of trophoblast organoid. *VCT*, Cytotrophoblast; *STB*, Syncytiotrophoblast (By Figdraw).

Due to the limited lifetime of TSCs *in vivo*, the self-renewal capacity of most sorted organ tissues will diminish over time. However, the study by Haider et al. discovered self-renewing vCTB precursors within CTB-ORGs through molecular analysis. These cells rely on Wnt signaling and have the ability to fuse and generate Notch1-positive EVT precursors after Wnt stimulation is lost, making them bipotent CTB stem cells. The model successfully replicates the development of trophoblast progenitors and different subtypes, including STB and EVT, in a 3D environment. Its ability to grow and differentiate under precise culture conditions makes it a valuable tool for modeling placental diseases and studying trophoblast development and function. Figure 3 briefly depicts a model diagram of their constructed trophoblast organoids.

Both trophoblast cells and decidual tissues play crucial roles in maternal and fetal immune responses. While various models and organoids derived from uterine glands have been utilized to simulate different physiological processes, there remains a dearth of innate infection models. To investigate their specific contributions to virus resistance, Yang et al. (2022) developed trophoblast cells and decidual tissue organoids to mimic the HCMV virus model. The reactivity of these two types of organs to HCMV infection was assessed, along with their observed differences. Furthermore, a co-culture technique was employed to examine the interaction between trophoblast cells and decidual epithelial cells. This model offers a straightforward and accessible approach to explore the interconnected responses among distinct pregnancy tissues.

At present, a study conducted by Karvas et al. (2022) in 2022 introduced a new model that combines stem cells and organoids, specifically TSCs derived from immature hPSCs that spontaneously form trophoblast organoids (SC-TOs) with villus structures similar to primary trophoblast organoids. The study utilized single-cell transcriptome analysis to identify distinct clusters of CTB, STB, and EVT, which are closely associated with trophoblast identity in the postimplantation embryo. Additionally, the study revealed that these organoid cultures exhibited the clonal X chromosome inactivation pattern observed in human placenta (Andoh-Noda et al., 2017) and showed selective susceptibility to emerging pathogens such as SARS-CoV-2 and Zika virus, which correlated with the expression levels of their respective entry factors. The SC-TOs provide an accessible 3D model system for studying the developing placenta and its susceptibility to emerging pathogens, offering comparable tissue architecture, placental hormone secretion, and long-term self-renewal capacity to previous trophoblast organoids. This study not only demonstrated the trophoblast identity and differentiation capacity of multiple cells in organoids but also characterized the X chromosome inactivation dynamics during the process from naive hiPSCs to organoid establishment. Furthermore, this model was utilized to explore placental development and susceptibility to different pathogens.

In the same year, Qin Jianhua’s team utilized biology and engineering technologies to establish a 3D model of hiPSCs-derived trophoblasts *in vitro*. This model was created using cell and organ chip technology, allowing for the simulation of developmental characteristics of early human placenta and the effects of hydrodynamic factors on placental tissue differentiation and secretion function (Cui et al., 2022). The researchers perfused hiPSCs in a fluid device, resulting in the formation of trophoblastic tissue in a biomimetic microenvironment. The device incorporated the extracellular matrix (ECM) and facilitated the generation of 3D clusters with major cell types of the human placenta, including trophoblast progenitors, STBs, and EVTs, through long-term 3D culture. RNA-seq analysis confirmed that the resulting CTB-ORGs exhibited phenotypic features highly similar to human first-trimester placental tissue. Further studies demonstrated that the organoids responded to stimulation by TNF-α and VEGF receptor inhibitors, effectively mimicking the physiological and pathological characteristics of placental tissue *in vivo*.

The advantage of their model is that, under dynamic culture conditions, the formed tissues exhibit enhanced expression of CTB, STB, and EVT-related markers at the gene and protein levels. RNA-seq analysis showed higher expression of trophoblast-specific genes in 3D tissues, indicating the important role of fluid flow in promoting trophoblast differentiation of hiPSCs. The established 3D placenta model combines bioengineering strategies with developmental principles and provides a comprehensive platform for studying placental biology in the biomimetic microenvironment of health and disease. It includes various trophoblast cell subtypes, vascular-like structures, and key functional characteristics of tissues, offering new insights into the study of human early placental development, preeclampsia, and pathogen infection.

### 4.3 Culture system of trophoblast organoids

In 2020, Sheridan et al. (2020) conducted a study on the limitations of current *in vitro* models of trophoblast cells and gained new insights into the cultivation of placental organoids. Moreover, the use of animal models poses inherent limitations in studying human placental development and function due to species variations. Consequently, creating precise *in vitro* models that accurately represent the distinct characteristics of the human placenta has been a significant hurdle.

The culture system used by Sheridan et al. (year) to extract organoids from early human placental specimens was based on existing organoid systems. In this method, cells isolated from tissues are encapsulated in Matrigel droplets supplemented with specific media (Sato et al., 2009). Through repeated refinement and validation, they established an optimized basic formulation for the growth of trophoblast cell organoids. The formulation includes EGF, hepatocyte growth factor (HGF), FGF-2 as a MAPK activator (Hempstock et al., 2004; Paiva et al., 2011; Kunath et al., 2014), CHIR99021 and R-Spinin-1 as Wnt pathway activators (Sonderegger et al., 2007), Y-27632 as a ROCK inhibitor (Shiokawa et al., 2002), PGE-2 as a cAMP/Akt pathway activator (Meadows et al., 2004), and A83-01 as a TGF-β inhibitor (Wu et al., 2017). Additionally, a simple enzymatic treatment with Accutase during culture significantly increased the expansion and proliferation of trophoblast cells (Lai et al., 2022).

CTB-ORGs are trophoblast cells isolated from first-trimester placentas and cultured in a 3D system to promote their long-term expansion as trophoblastic organoplasm. These organ tissues can be established within 2–3 weeks, passaged every 7–10 days, and maintained over a year. Under these optimized conditions, the organ tissue exhibits a villous structure composed of STB and CTB that have the ability to continuously proliferate. The structural arrangement of these human trophoblast organ tissues closely resembles that observed in villous placentas, with a layer of proliferating CTBs and the potential to differentiate into overlapping STB (Sheridan et al., 2020). Efficient generation of EVT was achieved by removing all growth factors and Wnt activators, inhibiting TGF-β, and introducing NRG1 (Maenhoudt et al., 2020). These EVT cells demonstrated rapid migration and invasion when placed in Matrigel droplets.

This organoid culture system offers a unique experimental model for studying the development, function, and interaction between trophoblast cells and the local and systemic maternal environment of the human placenta. It effectively addresses the limitations of previous models and allows for the exploration of various aspects of placental biology and pathology in a more physiological context.

### 4.4 Advantages and limitations of trophoblast organoids

This study provides a convenient and efficient system for studying various aspects of the placenta, including trophoblast differentiation pathways and interactions with other cell types. Additionally, 3D organoid models offer significant advantages over 2D cell culture as they allow for the modeling of specific cell-cell interactions and mechanical cues that arise from the structural complexity during early placental formation. Moreover, the creation of CTB-ORGs opens up opportunities for clinical *in vitro* drug studies. In the future, investigating trophoblast cell-like organoid cultures under pathological pregnancy conditions, such as preeclampsia, could help us understand potential mechanisms associated with related diseases and advance our knowledge of these disorders. Table 2 presents a summary of the trophoblast organoid models discussed earlier, indicating their sources and differentiation directions. Additionally, it provides a brief overview of the advantages associated with each model. This allows researchers to choose different experimental models based on their specific experimental requirements.

**TABLE 2:** Emerging Trophoblastic Organoid Models.

Author	Date	Source	Direction of differentiation	Advantage	Trophoblastic specific criteria
Margherita Y. Turco et al.	2018	VCT	EVT	To study human placental development and to study the interaction between the local and systemic maternal environment of the trophoblast	Yes
Sandra Haider et al.	2018	CTB	STB and HLA-G-EVT	To summarize the formation of trophoblast progenitor cells and differentiated subtypes in the 3D direction	Yes
Jian-Hua Qin et al.	2022	hiPSC	Trophoblast progenitor cells, STB and EVT	3D placental models combine bioengineering strategies with developmental principles	Yes
Rowan M Karvas et al.	2022	Naïve hPSCs	CTB, STB and EVT	An accessible 3D model system of the developing placenta and its susceptibility to emerging pathogens is provided	Yes

However, it is important to acknowledge the limitations and shortcomings of CTB-ORGs. One notable challenge is the misplacement of CTB and STB in organoids compared to placental villus. CTB spontaneously fuse in the center of organoids to form ENDOU, GCM1, and hCG^+^ STB, while there is a gradual decrease in cell proliferation from the outer edges of the organoids to the center. Overcoming this problem would open up more possibilities for studying the initiation of syncretization of trophoblast cells and uncovering the physiological functions of the placenta. Additionally, it should be noted that current trophoblast cell-like organoid tissues are exclusively derived from primordial placental tissue, which raises ethical and legal concerns and limits their applicability in potential diseases. Therefore, in order to broaden the scope and relevance of trophoblast cell organoid research, further investigations on the utilization of whole placental tissue to create similar organoids are necessary, although this will undoubtedly pose a difficult challenge.

## 5 Placenta explants

Explant from the human placenta is commonly utilized for studying cell proliferation and differentiation (Orendi et al., 2011), as well as placental transporters and metabolic enzyme studies (Lee et al., 2013). These explants are typically categorized into two groups. Explants from normal pregnancies are employed to investigate the impact of external factors on tissue function, as well as to compare normal pregnancy tissues of the same gestational age with explants from placentas affected by known pathologies (e.g., preeclampsia and intrauterine growth restriction) (Miller et al., 2005; Mehendale et al., 2007). While both early and late placentas can be used to study functions related to chorionic trophoblast cells, the early placenta cultures are specifically used to investigate chorionic EVT differentiation and invasion as they better maintain complete functionality. Additionally, unlike primary cells, the placental tissue used for culturing the explant cannot undergo freezing or thawing.

Depending on the purpose of the study, chorionic explants are cultured under different conditions. For instance, during early gestation, chorionic explants are typically cultured at the bottom of a well or embedded in an insert with a polycarbonate film underneath. On the other hand, during late gestation, chorionic explants are cultured at the bottom of a well, free-suspended, and then free-floating chorionic villus can be suspended in culture on styrene blocks or through a mesh support (Sooranna et al., 1999; Simán et al., 2001; Matalon et al., 2005; Toro et al., 2014).

*In vitro* culture of placental villus explants is commonly performed under static conditions. However, it is important to note that static placental villus explant culture differs significantly from the *in vivo* situation. *In vivo*, trophoblast cells invade the spiral arteries, leading to vascular end dilation. Additionally, free-floating placental villus adapt to fluid shear stresses caused by placental perfusion of maternal plasma and blood. Therefore, it is necessary to establish functional and indigenous chorionic villus explants. In a study by Kupper et al. (2021), a milder and simpler flow culture method for placental villus explants was established as an alternative to the commonly used static method. The study simulated the perfusion of the interchorionic space using an *in vitro* flow system, mimicking the intrauterine environment. The explant was positioned in a chamber to avoid direct exposure to the flow of medium and only passed through the top. The flow rate of the chorionic explant was set at 1 mL/min (Burton et al., 2009; Wang and Zhao, 2010). By analyzing the effect of the blood flow system on the activity and structural integrity of placental tissues using unbiased morphological and biochemical parameters, the study demonstrated the benefits of culturing tissues under flow conditions. The data showed a higher tendency of tissue disintegration in static cultured tissue compared to flow cultured tissue.

In both early and late pregnancy tissues, the STB undergoes progressive degeneration, believed to be driven by apoptosis (Palmer et al., 1997; Sooranna et al., 1999; Simán et al., 2001). However, Kupper et al. (2021) discovered that apoptosis increased in static cultured tissues after 48 h, but this degradation appeared to decrease or at least remain at the same level after flow culture. There are two advantages to flow-cultured explants: first, the placental vascular endothelial cells within the explants remain intact, and second, there are fewer vesicular structures on the chorionic surface of explants cultured under flow conditions. The use of placental explants cultured under flow conditions enables the analysis of the fetal-maternal interface, aiding in the understanding of various pregnancy pathologies such as preeclampsia. Additionally, it can be utilized for early pregnancy placental tissue culture and to simulate different pregnancy conditions by altering the experimental conditions.

## 6 Isolated placental perfusion models

Many studies involving the placenta cannot be directly conducted in humans due to ethical issues. Although animal experiments provide some reference significance, it is challenging to fully extrapolate their results to humans. To address this problem, the isolated placenta perfusion model was proposed. This model, initially suggested by Panigel et al. (1967), involves connecting the umbilical vein and umbilical artery supplying the same placental leaflet through a perfusion tube to establish fetal collateral circulation. Additionally, the perfusion tube is inserted into the stump of the spiral artery to establish maternal collateral circulation. This model has been further improved by Schneider et al. (1972) and Miller et al. (1985), eventually evolving into the mature isolated placenta perfusion model.

To preserve the intact placenta, we collected it for lavage. We ligated the umbilical vein that supplies the same placental leaflet to the branch of the umbilical artery. Then, we inserted tubes through puncture to establish fetal circulation in that placental leaflet. We ensured that the amount of perfusion at the end of the umbilical artery was equal to the amount of outflow at the end of the umbilical vein, indicating no leakage of the placental leaflet. Next, we removed the excess placental tissue and inserted two perfusion tubes 2–3 mm into the chorionic gap on the maternal side of the placenta to establish maternal circulation. Once the isolated placental perfusion model was established, we oxygenated the maternal-side perfusate with a mixture of 95% O_2_ and 5% CO_2_. Additionally, we inflated the fetus with a mixture of 95% N_2_ and 5% CO_2_ to simulate the *in vivo* state of maternal blood and fetal umbilical artery blood. Typically, the loss of fetal collateral perfusate does not exceed 2–4 mL/min, indicating the successful establishment of the cycle (Mathiesen et al., 2010).

*In vitro* placental perfusion models have been developed in various laboratories to facilitate research on the human placenta. These models have been used to study placental transport of nutrients and exogenous substances (Lee et al., 2018; Warth et al., 2019; Kurosawa et al., 2020), immune factors (Jain et al., 2014), trophoblast responses to infectious agents (Desforges et al., 2018), toxic substances (Bongaerts et al., 2021), and endogenous substances (Eshkoli et al., 2013). However, there are limitations to these models. The complexity of the perfusion system makes it difficult to establish and standardize, limiting widespread use. Additionally, although the isolated placenta is tested under near-physiological conditions, there are some differences compared to *in vivo* conditions, such as enzyme activity, number of transporters, and damage caused by ischemia and hypoxia during the preparation process. Despite these shortcomings, *ex vivo* placental perfusion remains a popular choice among researchers as the classical method to study placental function. Future studies should aim to gain a deeper understanding of the factors affecting *ex vivo* placental perfusion in order to develop a model that closely resembles the *in vivo* environment.

## 7 Comparison between trophoblast cell models and organoid models

CTB-ORGs and TSCs are valuable tools for gaining further insight into human placental development. Human trophoblast stem cells are obtained from the by-products of the blastocyst or early gestational placenta and can be cultured for extended periods or induced to differentiate into stem cell transplants or EVT. Additionally, trophoblast cell-like organs are derived from the placenta during early pregnancy and form chorionic structures comprised of proliferative VCTs that naturally transform into multinucleated STB. Under specific culture conditions, these organs readily promote differentiation into EVT. Both trophoblast-like organs and trophoblast stem cells originate from the chorionic cell trophoblast and differentiate into EVT or STB.

Both models have been reported to meet the following criteria for characterizing the trophoblast in early pregnancy *in vivo*: 1) expression of typical trophoblast markers; 2) unique features of human leukocyte antigen (HLA) class I molecules; 3) expression of microRNAs (miRNAs) from chromosome 19 cluster (C19MC); and 4) methylation of the ELF5 promoter. However, there are also notable differences between the two models, as reported by Megan A. Sheridan et al. TSCs resemble cells at the bottom of the cell column of EVT origin and do not readily undergo STB differentiation. On the other hand, organoids exhibit similarities to VCT and undergo spontaneous STB differentiation. A key characteristic of human trophoblast cells is that VCT and STB are human leukocyte antigen HLA null, while EVT expresses HLA-C, HLA-G, and HLA-E molecules. Trophoblast-like organoids retain these HLA expressions *in vivo*. Trophoblast stem cells, however, express only classical HLA-A and HLA-B molecules and maintain their expression even after EVT differentiation, while also upregulating HLA-G. Furthermore, the HLA expression of trophoblast stem cells in the 3D environment differs from that in the 2D environment. Therefore, trophoblast stem cells are more suitable for studies in the 2D environment, whereas trophoblast cell-like organoids are better suited for the 3D environment (Sheridan et al., 2021).

## 8 Conclusion and prospects

The human placenta is commonly used for *in vitro* studies due to its easy availability post-delivery. Various *in vitro* methods and experimental models have been established to investigate placental function. The trophoblast cell model and the trophoblast organoid model serve as effective *in vitro* models for studying the trophoblast and placenta from 2D and 3D perspectives, respectively. The acquisition of trophoblast stem cells not only provides a source for studying physiological changes in placental cells during pregnancy but also plays a crucial role in constructing cell and organoid models. Additionally, the establishment of placental trophoblast cellular organoids offers a reliable platform to explore maternal-fetal interactions during pregnancy and visualize physiological and pathological changes in 3D. Furthermore, the placenta explant and placenta isolated perfusion models serve as valuable *in vitro* models for studying the trophoblast and placenta from both 2D and 3D perspectives. These models, which closely mimic physiological conditions, have also significantly contributed to the study of various diseases’ physiological models.

Although many of them are organoid and stem cell models derived from trophoblast cells and chorionic villus explants, the advancement of technology will lead to a better understanding of the placenta as a transient organ. As a result, placental organoids and *in vitro* models of the placenta will become increasingly common and play a significant role in addressing various illnesses and challenges encountered during pregnancy.
